# Polycystic Ovary Syndrome (PCOS)-Specific Risk Appraisal of the Sunscreen Ultraviolet (UV) Filters (Oxybenzone/Octinoxate)

**DOI:** 10.3390/toxics13110927

**Published:** 2025-10-29

**Authors:** Sulagna Dutta, Pallav Sengupta, Bhupender S. Chhikara, Grzegorz Formicki, Israel Maldonado Rosas, Shubhadeep Roychoudhury

**Affiliations:** 1 Basic Medical Sciences Department, College of Medicine, Ajman University, Ajman 3464, United Arab Emirates; 2 Department of Biomedical Sciences, College of Medicine, Gulf Medical University, Ajman 4184, United Arab Emirates; 3 Department of Chemistry, Aditi Mahavidyalaya, University of Delhi, Delhi 110039, India; 4 Institute of Biology and Earth Sciences, University of the National Education Commission, 31-054 Krakow, Poland; 5 Citmer Reproductive Medicine, Mexico City 11520, Mexico; 6 Department of Life Science and Bioinformatics, Assam University, Silchar 788011, India

**Keywords:** endocrine disruptor, ethylhexyl methoxycinnamate, reproductive endocrinology, benzophenone-3, polycystic ovary

## Abstract

Polycystic ovary syndrome (PCOS) is a complex endocrine-metabolic disorder affecting 6–20% of women of reproductive age, manifesting through hyperandrogenism, ovulatory dysfunction, insulin resistance, and diverse metabolic derangements. Increasing evidence highlights the contribution of environmental factors, particularly endocrine-disrupting chemicals (EDCs), to PCOS susceptibility and severity. Sunscreen ultraviolet (UV) filters such as oxybenzone (benzophenone-3) and octinoxate (ethylhexyl methoxycinnamate) are widely used EDCs with established systemic absorption and biomonitoring evidence in human populations. Their endocrine-disrupting potential encompasses estrogenic and anti-androgenic activity, interference with steroidogenic enzymes, modulation of thyroid hormone, induction of oxidative stress, and epigenetic reprogramming, all of which are mechanistic pathways that overlap with PCOS pathophysiology. This evidence-based study critically appraises the evidence linking oxybenzone and octinoxate exposures to ovarian endocrinology, with a PCOS-specific focus. Human exposure patterns, pharmacokinetics, and regulatory perspectives are summarized alongside preclinical and in vitro data implicating these filters in ovarian dysfunction. Mechanistic intersections with PCOS include hyperandrogenism, disrupted folliculogenesis, oxidative stress-adipokine imbalance, and potential impairment of vitamin D signaling. Although epidemiological studies directly addressing PCOS outcomes remain sparse, the convergence of toxicological evidence with known endocrine vulnerabilities in PCOS underscores a need for targeted investigation. By mapping exposure pathways and mechanistic disruptions, this appraisal emphasizes the translational relevance of UV filter toxicity in the context of PCOS. It advocates for PCOS-specific biomonitoring cohorts, mechanistic studies, and regulatory consideration of reproductive endpoints while balancing the dermatological benefits of photoprotection against reproductive risks.

## 1. Introduction

Polycystic ovary syndrome (PCOS) is one of the most prevalent endocrine-metabolic disorders among women of reproductive age, with global prevalence estimates ranging from 6% to 20% depending on diagnostic criteria [1]. It is characterized by reproductive disturbances such as chronic anovulation, hyperandrogenism, and polycystic ovarian morphology (PCOM) [2], accompanied by systemic metabolic derangements including insulin resistance (IR), obesity, dyslipidemia, and low-grade inflammation [3,4]. These complex features underscore PCOS as a multifactorial condition influenced by both genetic and environmental determinants [5].

In recent years, emerging pollutants, particularly endocrine-disrupting chemicals (EDCs), have attracted increasing concern for their potential role in reproductive pathophysiology [6,7]. EDCs interfere with hormonal homeostasis through receptor interactions, enzyme modulation, or epigenetic reprogramming, thereby perturbing reproductive and metabolic functions [7]. Among these, sunscreen ultraviolet (UV) filters such as oxybenzone (benzophenone-3) and octinoxate (ethylhexyl methoxycinnamate) represent widely used chemicals that exhibit estrogenic, anti-androgenic, and thyroid-disrupting activities [8]. Due to their extensive use in personal care products and their detection in human biological fluids, these compounds warrant specific evaluation in relation to ovarian endocrinology [9]. The rationale for this appraisal is anchored in the convergence between the endocrine-disrupting properties of oxybenzone and octinoxate and the endocrine vulnerabilities observed in PCOS [9]. Perturbations in androgen-estrogen balance, steroidogenic enzyme function, oxidative stress, and adipokine-insulin signalling represent shared mechanistic domains [10,11,12]. Moreover, women of reproductive age, already the primary consumers of sunscreens, constitute the demographic most susceptible to PCOS, highlighting a critical overlap of exposure and risk [9]. Accordingly, this review aims to (a) summarize the current evidence on human exposure to oxybenzone and octinoxate, (b) delineate mechanistic pathways of endocrine disruption relevant to PCOS pathophysiology, and (c) discuss the translational and clinical implications of these exposures for reproductive health. This integrative analysis seeks to contextualize sunscreen UV filters within the broader landscape of environmental contributors to PCOS, thereby informing research, regulation, and clinical practice.

## 2. Human Exposure to Sunscreen UV Filters

### 2.1. Sources of Exposure

Human exposure to sunscreen UV filters such as oxybenzone and octinoxate occurs predominantly through dermal application, but secondary routes, including inhalation and ingestion, are increasingly recognized as relevant contributors to systemic burden [13]. Dermal absorption is the most common pathway, as these compounds are integral to lotions, creams, and cosmetic formulations intended for photoprotection [14]. Both oxybenzone and octinoxate possess lipophilic properties that facilitate their penetration through the stratum corneum into systemic circulation [15], a process enhanced by factors such as skin hydration, frequency of application, and use over large body surface areas. Repeated application during daily skincare routines and recreational sun exposure significantly elevates cumulative exposure [16].

Inhalation represents another route, particularly with spray-based sunscreens and aerosolized cosmetic products [17]. Experimental evidence has demonstrated that inhaled micro- and nanoparticles can deposit within the respiratory tract, leading to mucosal absorption and subsequent systemic distribution [18]. Although quantitative estimates of inhaled doses are less robust compared to dermal absorption, the potential for chronic low-dose exposure remains clinically relevant. Ingestion constitutes an indirect but noteworthy pathway [19]. Oxybenzone and octinoxate are increasingly detected in aquatic ecosystems due to wastewater runoff and direct contamination from recreational activities [20]. Bioaccumulation in fish and seafood introduces a dietary exposure pathway, while contaminated drinking water may serve as an additional source [21]. The persistence of these compounds in environmental matrices, coupled with their lipophilicity, raises concerns regarding chronic low-level ingestion in populations with high seafood consumption. Therefore, the multiplicity of exposure routes underscores the ubiquity of sunscreen UV filters in daily life. For women of reproductive age, especially those predisposed to PCOS, this constant background exposure may constitute an underappreciated environmental risk factor influencing ovarian endocrinology [11]. Recognition of these pathways provides the foundation for interpreting biomonitoring data and assessing dose–response relationships relevant to reproductive health outcomes.

### 2.2. Pharmacokinetics

The pharmacokinetic profiles of oxybenzone and octinoxate reveal their capacity for systemic exposure and bioactivity beyond cutaneous sites [22]. Dermal penetration is facilitated by their low molecular weight and lipophilic characteristics, allowing for traversal through epidermal barriers [23]. Clinical studies have confirmed measurable plasma concentrations following topical application [22,24], with systemic absorption exceeding thresholds initially presumed to be negligible. In fact, the United States Food and Drug Administration (FDA) reported that plasma levels after maximal-use sunscreen trials surpassed levels requiring formal toxicological evaluation [25].

Once absorbed, oxybenzone undergoes hepatic metabolism primarily via glucuronidation and sulfation, producing conjugated metabolites that circulate systemically and are excreted predominantly in urine [26]. Octinoxate follows a similar metabolic trajectory, although variability in biotransformation efficiency has been reported across individuals [27]. Despite relatively rapid metabolism, repeated use leads to sustained steady-state concentrations, given their re-application in cosmetic routines. Importantly, tissue distribution studies suggest potential accumulation in lipid-rich tissues, including the brain, liver, and adipose depots, raising concern for chronic low-level bioaccumulation [28]. The half-life of oxybenzone in systemic circulation has been reported between 4 and 24 h, whereas octinoxate demonstrates shorter plasma persistence but may accumulate in tissues with repeated exposure [29]. Notably, these compounds and their metabolites cross biological barriers, including the placenta, underscoring reproductive safety concerns [30]. Moreover, their ability to persist in follicular fluid, breast milk, and seminal plasma highlights systemic dissemination across multiple reproductive compartments [31]. Thus, the pharmacokinetics of oxybenzone and octinoxate challenge the conventional perception of sunscreens as inert topical agents [29]. Their systemic bioavailability, potential for tissue bioaccumulation, and transplacental passage warrant critical evaluation in the context of reproductive endocrinology. In women with PCOS, who already exhibit endocrine and metabolic dysregulation, such pharmacokinetic properties may exacerbate pathophysiological processes through sustained endocrine-disrupting influences.

### 2.3. Biomonitoring Evidence

Biomonitoring studies have provided compelling evidence of widespread human exposure to sunscreen UV filters, particularly oxybenzone, with urinary concentrations serving as reliable biomarkers of internal dose [32]. Large-scale surveys, such as the United States National Health and Nutrition Examination Survey (NHANES), have consistently reported detection rates exceeding 95% in the general population, underscoring ubiquitous exposure [33]. Median urinary oxybenzone levels often vary by demographic factors, reflecting differences in product use, lifestyle, and environmental contamination [34,35].

Sex-based differences are especially prominent, with women exhibiting significantly higher urinary concentrations than men, attributable to greater use of personal care and cosmetic products containing UV filters [36]. Age stratification further reveals elevated exposures among adolescents and young adults, coinciding with higher cosmetic consumption and lifestyle-related behaviours [37]. Geographic variability is also marked: populations in regions with higher ambient UV indices and cultural practices favouring sunscreen use demonstrate elevated biomonitoring levels [38]. Conversely, dietary exposure through contaminated seafood contributes disproportionately to coastal populations reliant on marine food sources [39].

Beyond population averages, biomonitoring has detected extreme outliers with urinary oxybenzone levels exceeding toxicological reference values, raising concern about high-exposure subgroups. Importantly, reproductive-aged women, the demographic most affected by PCOS, are consistently among the highest-exposed cohorts, highlighting a potential environmental intersection with reproductive health vulnerability [40]. Emerging studies have extended biomonitoring beyond urine to matrices such as breast milk, amniotic fluid, and follicular fluid, reinforcing evidence of systemic dissemination and reproductive compartment penetration [31,34]. However, PCOS-specific biomonitoring cohorts remain absent, representing a significant gap in exposure science. Given that PCOS women often demonstrate altered xenobiotic metabolism and adipose distribution, extrapolation from general population data may underestimate the risk in this subgroup. Thus, biomonitoring evidence not only confirms ubiquitous exposure but also frames the urgent need for PCOS-focused toxicological surveillance.

### 2.4. Regulatory and Toxicological Thresholds

The regulatory landscape surrounding oxybenzone and octinoxate reflects ongoing debate regarding their safety profiles, particularly in relation to systemic absorption and endocrine activity. In the United States, the FDA has historically classified both compounds as Category I (generally recognized as safe and effective) under sunscreen monographs [41]. However, recent maximal-use clinical trials have demonstrated systemic concentrations exceeding the threshold (0.5 ng/mL) requiring comprehensive toxicological evaluation [42]. Consequently, the FDA has called for additional safety data on oxybenzone and octinoxate before reaffirming their regulatory status [41]. In contrast, the European Union adopts a more precautionary stance. The Scientific Committee on Consumer Safety (SCCS) has established maximum allowable concentrations of 6% for oxybenzone and 10% for octinoxate in cosmetic formulations, with ongoing review of data on endocrine disruption [43]. Certain European Union (EU) member states have also supported restrictions based on environmental persistence and ecotoxicological concerns. Similarly, regulatory authorities in Asia demonstrate heterogeneity: Japan permits oxybenzone use up to 10% [44,45], whereas China maintains broader allowances under its cosmetic safety framework. Notably, countries such as Thailand and South Korea are revisiting regulatory thresholds in light of concerns over ecological toxicity and human health [46]. Beyond concentration limits, regional bans exemplify shifting policy priorities. Hawaii and Palau have prohibited the sale of sunscreens containing oxybenzone and octinoxate due to environmental damage to coral reefs, thereby indirectly highlighting concerns about human exposure [47]. However, regulatory frameworks seldom incorporate PCOS-specific endpoints, relying instead on generalized reproductive toxicity assays. Overall, regulatory thresholds remain variably aligned with emerging toxicological evidence. While progress has been made in acknowledging systemic absorption, current frameworks inadequately account for endocrine vulnerabilities in at-risk populations such as women with PCOS. Integration of disease-specific endpoints into chemical risk assessments represents a critical unmet regulatory need.

## 3. Mechanistic Basis of Endocrine Disruption by Oxybenzone & Octinoxate

### 3.1. Estrogenic and Anti-Androgenic Action

Oxybenzone and octinoxate are among the most studied UV filters with demonstrable endocrine activity through interactions with nuclear hormone receptors [8] (Table 1). Oxybenzone exhibits weak but biologically relevant estrogenic activity by binding to estrogen receptors (ERα and ERβ) and functioning as a partial agonist [48]. This mimetic effect, although less potent than endogenous estradiol, may cumulatively disrupt estrogen-regulated signalling, particularly under conditions of chronic low-dose exposure [31,48]. Octinoxate similarly demonstrates estrogenic activity, though with lower receptor affinity, and has been shown to activate ER-dependent transcription in cell-based assays [49]. Beyond estrogenic mimicry, both compounds exert anti-androgenic effects by competing with androgen for binding to the androgen receptor (AR). By blocking androgen-mediated transcriptional activity, oxybenzone and octinoxate may impair androgen-responsive pathways critical for ovarian folliculogenesis and steroidogenesis [48]. Such dual activity, weak estrogen agonism combined with AR antagonism, creates a complex endocrine milieu characterized by disrupted estrogen-androgen homeostasis [48,49]. Beyond receptor interactions and interference with hormone synthesis, EDCs may disturb endocrine balance through a more subtle route: competition for serum binding proteins. By binding to carriers like sex hormone–binding globulin (SHBG) and thyroxine-binding globulin (TBG), these compounds can displace native hormones from their transport sites. Even minor shifts in this equilibrium may alter the proportion of free, biologically active hormones in circulation. Such disruption could intensify hormonal irregularities already characteristic of PCOS, adding another layer to its complex endocrine profile [50].

This imbalance is of particular relevance to PCOS, where intrinsic hyperandrogenism already drives reproductive and metabolic abnormalities. Additional exogenous disruption through UV filters could potentiate follicular arrest, ovulatory dysfunction, and reproductive hormone imbalance.

Animal studies further corroborate receptor-mediated disruption, with reported outcomes including altered uterine weight, irregular estrous cyclicity, and impaired fertility [58]. In vitro experiments with human cell lines reveal transcriptional changes in estrogen-responsive genes and suppression of androgen-induced signalling, reinforcing their endocrine-disrupting potential [52]. Importantly, receptor binding activity occurs at concentrations overlapping with those detected in human biomonitoring studies, underscoring translational relevance [40]. Thus, the estrogenic and anti-androgenic properties of oxybenzone and octinoxate establish a mechanistic basis for ovarian endocrine disruption [48]. For women with PCOS, characterized by heightened sensitivity to androgen-estrogen imbalance, such receptor-level perturbations may exacerbate underlying pathophysiology and influence reproductive outcomes.

### 3.2. Steroidogenic Enzyme Interference

**Figure 1:**
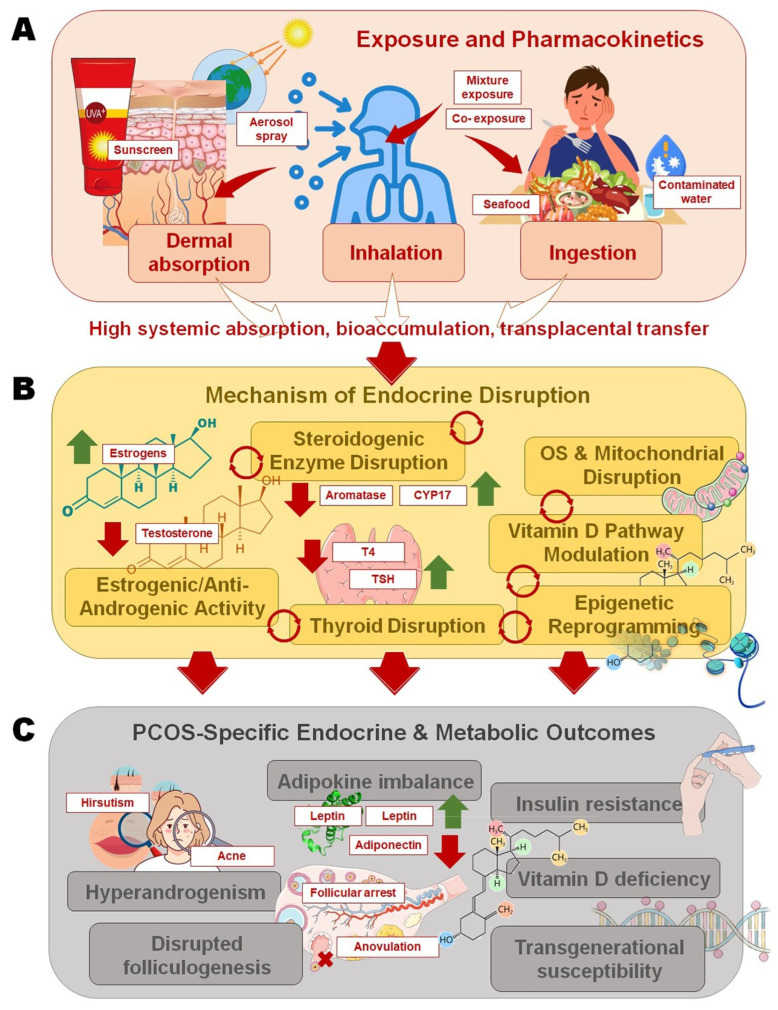
Pathways linking sunscreen ultraviolet (UV) filters to polycystic ovary syndrome (PCOS)-specific outcomes: (**A**) Oxybenzone and octinoxate enter the body through dermal absorption, inhalation of sprays, or ingestion of contaminated seafood and water, with high systemic absorption, bioaccumulation, and transplacental transfer; (**B**) Mechanistic disruptions include steroidogenic enzyme interference, estrogenic/anti-androgenic receptor activity, thyroid hormone imbalance, oxidative stress with mitochondrial dysfunction, vitamin D pathway modulation, and epigenetic reprogramming; (**C**) These pathways converge on PCOS pathophysiology, leading to hyperandrogenism, disrupted folliculogenesis and anovulation, adipokine imbalance with insulin resistance (IR), vitamin D deficiency, and potential transgenerational susceptibility.

In addition to receptor-mediated actions, oxybenzone and octinoxate directly interfere with steroidogenic enzyme networks, which are critical regulators of ovarian hormone biosynthesis. Aromatase (CYP19A1), which catalyzes the conversion of androgens to estrogens, is a key target [8]. Evidence from in vitro granulosa cell models shows that oxybenzone suppresses aromatase activity, leading to impaired estradiol synthesis [59]. Such inhibition disrupts the delicate balance between androgens and estrogens within the ovarian follicle, a hallmark derangement in PCOS pathophysiology (Figure 1).

Octinoxate has also been reported to inhibit steroidogenic acute regulatory protein (StAR) expression and downstream enzymes, including CYP17A1 and 3β-hydroxysteroid dehydrogenase (3β-HSD) [8,53]. These enzymes are central to androgen biosynthesis, and their dysregulation alters both ovarian and adrenal steroid output [60]. Experimental studies in rodent models exposed to octinoxate demonstrated increased ovarian testosterone levels accompanied by reduced estradiol, consistent with disrupted steroidogenic enzyme function [8]. Mechanistically, enzyme interference may arise from direct inhibition of catalytic activity or transcriptional repression via nuclear receptor crosstalk. For instance, oxybenzone-mediated suppression of aromatase may occur through estrogen receptor-dependent feedback mechanisms [59]. Such alterations in enzymatic flux shift ovarian steroid balance toward androgen excess, a defining feature of PCOS.

In clinical terms, the overlap between enzyme disruption and PCOS steroidogenic abnormalities highlights a potential exacerbating role of UV filters [9]. PCOS is characterized by increased CYP17 activity, leading to androgen excess [61]; exogenous compounds that further dysregulate aromatase or 3β-HSD amplify this imbalance [62]. This mechanistic overlap underscores the plausibility of UV filter exposure aggravating hyperandrogenism and ovulatory dysfunction in PCOS women. Ultimately, interference with steroidogenic enzymes provides a biochemical pathway linking UV filters to ovarian dysfunction. Future studies must delineate concentration thresholds and chronic exposure effects to determine whether observed in vitro and preclinical outcomes translate into measurable risks for PCOS populations.

### 3.3. Thyroid Hormone Disruption and Ovarian Dysfunction

Thyroid hormones play an integral role in reproductive physiology, with effects on folliculogenesis, steroidogenesis, and ovulatory regulation [63]. Disruption of thyroid function by environmental chemicals may therefore indirectly influence ovarian endocrinology [64]. Both oxybenzone and octinoxate have been implicated in thyroid hormone perturbation, primarily through interference with thyroid hormone synthesis, binding, and metabolism [27]. Animal studies reveal that oxybenzone exposure leads to reduced serum thyroxine (T4) levels and altered thyroid histology, suggestive of hypothyroid-like effects [27,65]. Similarly, octinoxate has been shown to suppress thyroid peroxidase (TPO) activity and modulate thyroid-stimulating hormone (TSH) concentrations. These disruptions can shift the hypothalamic-pituitary-thyroid (HPT) axis equilibrium, leading to compensatory hypersecretion of TSH and downstream metabolic derangements [54].

Thyroid dysfunction has established links with ovarian impairment, including menstrual irregularities, anovulation, and subfertility. In PCOS, subtle thyroid abnormalities are frequently documented, including a higher prevalence of subclinical hypothyroidism and autoimmune thyroiditis [66]. Disruption of thyroid homeostasis by UV filters could therefore exacerbate pre-existing vulnerabilities, amplifying the risk of anovulation and metabolic complications [27]. Moreover, thyroid hormone crosstalk with gonadotropins and insulin signalling is critical in ovarian function, pathways already disrupted in PCOS. Human epidemiological data, though limited, indicate associations between urinary oxybenzone levels and altered thyroid hormone profiles, particularly in women [27]. These findings align with experimental evidence and raise concerns about their real-world relevance. Given that PCOS is characterized by delicate endocrine balances, exogenous thyroid disruption may act as a secondary but significant contributor to reproductive dysfunction. Thus, UV filter-induced thyroid hormone disruption represents an indirect yet clinically meaningful pathway linking environmental exposure to ovarian dysfunction [67]. For PCOS populations, this interaction may worsen reproductive and metabolic manifestations, reinforcing the need for integrative assessments of thyroid endpoints in toxicological evaluations.

### 3.4. Oxidative Stress Induction and Mitochondrial Perturbation

Oxybenzone and octinoxate have been shown to induce oxidative stress and mitochondrial dysfunction [45]—both central mechanisms implicated in the pathophysiology of PCOS. Oxidative stress arises from an imbalance between reactive oxygen species (ROS) production and antioxidant defenses, resulting in cellular and molecular damage [55,68,69]. Several in vitro and animal studies demonstrate that exposure to these UV filters elevates ROS levels [70], depletes glutathione, and enhances lipid peroxidation in reproductive tissues. Mitochondrial perturbations constitute a parallel pathway of toxicity. Both oxybenzone and octinoxate disrupt mitochondrial membrane potential, impair ATP synthesis, and trigger cytochrome c release, indicative of apoptosis initiation [8]. In granulosa and theca cells, mitochondrial dysfunction compromises energy supply for steroidogenesis, leading to impaired follicular maturation [71]. This mechanistic pathway closely parallels observations in PCOS, where oxidative stress and mitochondrial dysfunction contribute to IR, chronic inflammation, and poor oocyte quality [55]. Moreover, oxidative stress induced by UV filters activates redox-sensitive signalling pathways such as NF-κB and MAPK [72], promoting pro-inflammatory cytokine release. In PCOS, such inflammatory amplification further disrupts ovarian microenvironments, aggravating follicular arrest and endocrine imbalance [73]. Importantly, oxidative stress also influences adipokine secretion patterns, linking UV filter toxicity to systemic metabolic dysfunction relevant to PCOS phenotypes.

The intersection of oxidative stress and mitochondrial injury is particularly concerning in the context of transgenerational effects [74]. Animal models suggest maternal UV filter exposure can induce oxidative damage in oocytes, potentially programming reproductive dysfunction in offspring [56,75]. For PCOS, a condition already associated with increased oxidative burden, additional environmental triggers such as oxybenzone and octinoxate may accelerate disease progression and complicate therapeutic outcomes. Overall, oxidative stress and mitochondrial perturbation provide a unifying mechanistic bridge between UV filter toxicity and PCOS pathology. These findings highlight the importance of evaluating redox biomarkers and mitochondrial endpoints in exposure studies.

### 3.5. Epigenetic Reprogramming

Beyond direct hormonal and oxidative effects, oxybenzone and octinoxate exert long-term influences through epigenetic reprogramming [13,76]. Epigenetic modifications, such as DNA methylation, histone modification, and microRNA (miRNA) regulation, serve as critical mediators linking environmental exposures to changes in gene expression without altering the DNA sequence [77]. Experimental studies suggest that exposure to oxybenzone alters the DNA methylation profiles of hormone receptor genes, potentially reprogramming estrogen and androgen responsiveness [78]. Octinoxate has been shown to influence histone acetylation and methylation states, modifying chromatin accessibility and transcription of genes involved in steroidogenesis and oxidative stress defence [57]. Such persistent alterations may underlie reproductive toxicity even after cessation of exposure. miRNA modulation provides another pathway of disruption. Oxybenzone exposure has been linked to altered expression of miRNAs [65] involved in ovarian follicle growth, apoptosis, and insulin signalling. Dysregulated miRNAs may silence key regulators of granulosa cell function and steroidogenic enzyme networks [79], thereby amplifying PCOS-like ovarian phenotypes.

Epigenetic modifications are particularly relevant in reproductive contexts due to their potential heritability. Animal studies suggest maternal exposure to UV filters can reprogram offspring reproductive outcomes via transgenerational epigenetic inheritance [30]. In PCOS, where intrinsic epigenetic dysregulation of steroidogenic and metabolic genes has been documented [80], exogenous epigenetic modifiers, such as UV filters, could worsen disease expression or increase susceptibility in subsequent generations. Thus, epigenetic reprogramming represents a critical mechanistic layer by which oxybenzone and octinoxate may contribute to PCOS pathophysiology. Unlike transient hormonal changes, epigenetic alterations may persist long-term, embedding exposure history into ovarian gene networks [81]. This highlights the necessity of incorporating epigenomic analyses into biomonitoring and toxicological studies of sunscreen UV filters.

## 4. Ovarian Endocrinology and PCOS Pathophysiology

### 4.1. Normal Ovarian Hormone Regulation

The ovarian endocrine milieu is orchestrated by the hypothalamic-pituitary-ovarian (HPO) axis, a finely tuned neuroendocrine network that governs reproductive function [82]. Gonadotropin-releasing hormone (GnRH), secreted in a pulsatile manner from the hypothalamus, stimulates the anterior pituitary to release follicle-stimulating hormone (FSH) and luteinizing hormone (LH) [83]. These gonadotropins act on ovarian granulosa and theca cells, respectively, to regulate folliculogenesis and steroidogenesis [84]. FSH promotes follicle growth and estradiol production via granulosa cell aromatase activity, while LH drives androgen synthesis in theca cells through CYP17A1 activation [85]. The “two-cell, two-gonadotropin” model underscores the interdependence of these processes, with granulosa-derived estradiol feeding back to modulate GnRH pulsatility and gonadotropin secretion [86]. Progesterone secretion by the corpus luteum during the luteal phase ensures endometrial receptivity and pregnancy maintenance [87]. This cyclical endocrine interplay is reinforced by negative and positive feedback loops involving estradiol, progesterone, inhibin, and activin [88]. Metabolic hormones such as insulin, leptin, and adiponectin further integrate energy balance with reproductive function, reflecting the broader metabolic sensitivity of ovarian physiology [89].

Disruption at any level of the HPO axis, hypothalamic signalling, pituitary gonadotropin release, ovarian steroidogenesis, or metabolic crosstalk, can impair ovulation and fertility [90]. Environmental stressors, including EDCs such as oxybenzone and octinoxate, have the potential to disturb this delicate equilibrium through receptor binding, steroidogenic enzyme interference, and cross-talk with metabolic pathways [31]. Understanding normal ovarian regulation, therefore, provides a critical baseline against which pathological perturbations, such as those observed in PCOS, can be contextualized.

### 4.2. PCOS Ovarian Features

PCOS is characterized by a constellation of ovarian and systemic features, with hyperandrogenism representing the central endocrine abnormality [91]. Excess androgen production arises primarily from dysregulated theca cell steroidogenesis, driven by heightened CYP17A1 activity and exacerbated by hyperinsulinemia. Elevated intraovarian androgen levels disrupt granulosa cell aromatase activity, impairing estradiol synthesis and perpetuating anovulation [92]. This endocrine imbalance manifests clinically as hirsutism, acne, and alopecia, alongside menstrual irregularities [91]. Disrupted folliculogenesis is another hallmark. While early follicle recruitment is intact, excess androgens and aberrant gonadotropin signalling prevent the selection of a dominant follicle, leading to follicular arrest and the characteristic PCOM [93]. Apoptotic changes in granulosa cells and impaired FSH responsiveness further compromise follicle maturation [94].

IR plays a dual role, both exacerbating hyperandrogenism via insulin-mediated LH receptor upregulation in theca cells and promoting systemic metabolic dysfunction [95]. The associated hyperinsulinemia synergizes with LH to drive androgen excess, creating a self-perpetuating loop [96]. Adipokine dysregulation, marked by elevated leptin, reduced adiponectin, and altered visfatin, further aggravates IR and ovarian dysfunction [97]. Vitamin D deficiency, commonly reported in PCOS populations, compounds this endocrine-metabolic imbalance [98]. Vitamin D modulates ovarian follicle development, steroidogenesis, and insulin sensitivity through vitamin D receptor-mediated pathways [99]. Deficiency is associated with worsened hyperandrogenism, impaired ovulation, and metabolic derangements, underscoring its role as a cofactor in PCOS pathophysiology [100]. Thus, the ovarian features of PCOS represent a nexus of hyperandrogenism, disrupted folliculogenesis, and metabolic imbalance, forming a vulnerable landscape where exogenous endocrine disruptors such as oxybenzone and octinoxate may exert compounding effects [91].

### 4.3. PCOS Heterogeneity (Phenotypes A–D) and Environmental Susceptibility

PCOS is a heterogeneous disorder with variable clinical and biochemical presentations, classified into four phenotypes under the Rotterdam criteria [2]. Phenotype A, the ‘classic’ form, combines hyperandrogenism, ovulatory dysfunction, and PCOM, representing the most severe metabolic and reproductive profile. Phenotype B includes hyperandrogenism and ovulatory dysfunction without PCOM, while phenotype C involves hyperandrogenism with PCOM but preserved ovulation. Phenotype D, the mildest, exhibits ovulatory dysfunction and PCOM in the absence of hyperandrogenism [101]. This phenotypic diversity underscores the multifactorial nature of PCOS, shaped by genetic, metabolic, and environmental interactions [5]. Women with phenotypes A and B are more prone to IR, obesity, and cardiometabolic risk, whereas phenotypes C and D often present with milder endocrine and metabolic abnormalities [101]. Importantly, environmental susceptibility appears to be uneven across phenotypes, with hyperandrogenic forms likely being more sensitive to endocrine-disrupting exposures. Exogenous estrogenic and anti-androgenic chemicals may amplify existing androgen-estrogen imbalance, while oxidative stress-inducing pollutants could exacerbate IR and follicular dysfunction [102].

Epidemiological studies suggest geographic and lifestyle variations in phenotype distribution, influenced by diet, physical activity, and environmental exposures [51]. For example, higher prevalence of phenotype A has been documented in South Asian and Middle Eastern populations, coinciding with elevated background exposure to environmental contaminants and higher metabolic risk [103]. This intersection raises the possibility that EDCs, including UV filters, may contribute to the phenotypic expression or severity of certain conditions. The heterogeneity of PCOS also complicates the assessment of risk. Current toxicological frameworks rarely stratify by phenotype, yet differential susceptibility could influence clinical outcomes. A precision-medicine perspective that integrates phenotype classification with environmental exposure profiling may therefore be essential. For oxybenzone and octinoxate, evaluating their endocrine-disrupting potential across distinct PCOS phenotypes could reveal differential vulnerabilities, informing risk appraisal and clinical guidance for women of reproductive age.

## 5. Linking UV Filters to PCOS-Specific Mechanisms

### 5.1. Hyperandrogenism: Evidence of Alteration in Androgen-Estrogen Balance by Oxybenzone/Octinoxate

Hyperandrogenism is a central feature of PCOS, driving both reproductive and metabolic dysfunction [61]. Evidence suggests that exposure to oxybenzone and octinoxate may exacerbate androgen-estrogen imbalance [104] through dual mechanisms: estrogenic receptor activation and anti-androgenic interference [8]. Oxybenzone binds to estrogen receptors with partial agonist activity, potentially amplifying estrogen-responsive pathways, whereas both compounds exhibit antagonism at the androgen receptor, diminishing androgen signalling [48]. Paradoxically, in ovarian tissues, suppression of aromatase activity by these compounds impairs androgen-to-estrogen conversion, resulting in elevated intraovarian androgen concentrations [48]. Preclinical studies lend weight to this disruption. Oxybenzone exposure in rodent models has been associated with increased serum testosterone and reduced estradiol, consistent with aromatase inhibition [31]. Similarly, octinoxate-treated animals demonstrate heightened ovarian androgen levels and disrupted estrous cyclicity [40]. These mechanistic findings parallel the intrinsic steroidogenic abnormalities in PCOS, where increased CYP17 activity drives excess androgen biosynthesis and insufficient aromatase activity fails to counterbalance it.

Human biomonitoring further raises concern. Elevated urinary oxybenzone levels have been correlated with altered sex hormone profiles in women, including higher testosterone-to-estradiol ratios [105]. While PCOS-specific datasets remain sparse, the overlap between known toxicological effects of UV filters and characteristic endocrine patterns of PCOS highlights a plausible aggravating influence. For PCOS women, whose baseline physiology already favours hyperandrogenism, exogenous disruption from sunscreen UV filters may intensify clinical symptoms such as hirsutism, acne, and menstrual irregularity. This mechanistic convergence highlights the importance of targeted biomonitoring in PCOS cohorts and raises questions about the cumulative endocrine burden resulting from lifestyle-associated exposures.

### 5.2. Insulin Resistance: Oxidative Stress and Adipokine Dysregulation as Mediators

IR is a defining metabolic disturbance in PCOS, implicated in both hyperandrogenism and cardiometabolic comorbidities [95,96]. Oxybenzone and octinoxate may indirectly exacerbate IR through the induction of oxidative stress and adipokine dysregulation [106]. Studies demonstrate that both compounds increase ROS generation, deplete antioxidant reserves such as glutathione, and activate redox-sensitive inflammatory pathways including NF-κB [74,107]. These processes impair insulin receptor signalling in adipose and muscle tissue, a mechanism paralleled in PCOS pathophysiology.

Adipokine imbalance is another critical mediator. PCOS women typically exhibit elevated leptin and resistin levels with reduced adiponectin, fostering IR and inflammation [108,109]. Animal studies reveal that UV filter exposure perturbs adipokine secretion, with oxybenzone exposure linked to altered leptin signalling and octinoxate associated with changes in adiponectin gene expression [110]. Such modifications exacerbate insulin signalling defects, particularly in the ovarian microenvironment, where insulin acts synergistically with LH to drive theca cell androgen overproduction [95]. The interplay between oxidative stress, adipokine imbalance, and IR represents a vicious cycle [111]. UV filter-induced ROS not only worsens insulin signalling but also promotes adipose inflammation, further deranging adipokine profiles. In PCOS, where oxidative stress and metabolic inflammation are already heightened, exogenous disruption compounds intrinsic metabolic vulnerability [55]. Clinical extrapolation suggests that women with PCOS exposed to higher systemic levels of oxybenzone and octinoxate may be predisposed to worsened IR, with implications for fertility, gestational outcomes, and long-term cardiometabolic risk [4]. Integrating oxidative and adipokine markers into exposure studies could clarify these links and support targeted interventions.

### 5.3. Folliculogenesis and Ovulatory Dysfunction

Normal folliculogenesis requires coordinated granulosa-theca cell interactions under the regulation of FSH and LH [86]. In PCOS, this process is disrupted, leading to follicular arrest and chronic anovulation [93]. Experimental data suggest that oxybenzone and octinoxate can exacerbate these disruptions through direct effects on granulosa cell viability and gonadotropin signalling [81]. In vitro studies have shown that oxybenzone induces apoptosis in granulosa cells, mediated by mitochondrial dysfunction and activation of caspase cascades [94]. Such cell death compromises estrogen synthesis and follicular maturation, key processes already impaired in PCOS. Octinoxate exposure has similarly been linked to reduced FSH receptor expression and impaired cyclic AMP signalling in granulosa cells, further weakening the ovarian response to gonadotropin stimulation [11]. Disruption of LH signalling is another potential mechanism. By interfering with steroidogenic enzyme expression and androgen synthesis, UV filters alter theca cell response to LH, leading to excessive androgen accumulation. This hormonal environment impedes granulosa cell aromatization of androgens to estrogens, reinforcing follicular arrest [85,112,113].

Animal models provide corroborative evidence, where oxybenzone exposure results in PCOM, increased follicular atresia, and irregular estrous cycles [114]. These findings parallel the histopathological features of PCOS ovaries, suggesting that UV filter exposure may not only worsen ovulatory dysfunction but also mimic PCOS-like phenotypes. For women with PCOS, already predisposed to chronic anovulation, exogenous disruption of granulosa cell survival and FSH/LH signalling may intensify infertility risk and reduce assisted reproduction outcomes [115]. Recognizing these links is critical for assessing environmental contributions to reproductive dysfunction.

### 5.4. Vitamin D Signalling Impairment

Vitamin D deficiency is prevalent among women with PCOS and is associated with worsened hyperandrogenism, IR, and impaired folliculogenesis [98]. Sunscreen use itself is known to reduce cutaneous synthesis of vitamin D3 by blocking UVB-mediated conversion of 7-dehydrocholesterol to cholecalciferol. While photoprotection is essential, chronic suppression of vitamin D synthesis poses potential endocrine consequences, particularly for PCOS populations [106]. Oxybenzone and octinoxate add further complexity by their systemic bioavailability and possible interference with vitamin D signalling pathways [106,116]. Evidence suggests that these compounds can alter nuclear receptor activity, including vitamin D receptor (VDR) cross-talk with estrogen and androgen receptors. Disruption of VDR-mediated transcription could impair ovarian steroidogenesis and granulosa cell function, both of which are critical for ovulation. Moreover, vitamin D modulates insulin sensitivity and adipokine balance, meaning its deficiency exacerbates PCOS metabolic features [117].

Population studies consistently report lower circulating 25-hydroxyvitamin D levels in women with PCOS compared to controls [118,119]. The contribution of sunscreen-derived UV filters to this deficiency has not been directly studied; however, chronic photoprotection combined with chemical interference in VDR pathways may amplify the risk. For women who are already deficient, the additive effect of UV filters could exacerbate reproductive and metabolic outcomes [106]. Thus, the intersection of sunscreen UV filter exposure, vitamin D deficiency, and PCOS pathophysiology warrants attention. Balancing photoprotection with strategies to maintain adequate vitamin D, through supplementation or alternative formulations, becomes particularly relevant for PCOS management. Research directly examining UV filter exposure, vitamin D signalling, and ovarian function in PCOS cohorts is urgently needed.

### 5.5. Epigenetic Effects: Potential Programming of PCOS Susceptibility in Offspring

Epigenetic mechanisms link environmental exposures to long-term reproductive outcomes [80], with growing evidence that oxybenzone and octinoxate can alter DNA methylation, histone modifications, and microRNA expression. Such changes are particularly concerning in the reproductive context, where germ cell and embryonic epigenomes are highly plastic [65,78]. Experimental data demonstrate that maternal exposure to oxybenzone alters the methylation of genes involved in steroidogenesis and folliculogenesis [120], while octinoxate influences histone acetylation patterns, affecting ovarian gene expression [57]. These modifications persist beyond direct exposure, raising the possibility of transgenerational inheritance. MicroRNA dysregulation has also been observed, with UV filter exposure linked to altered expression of miRNAs controlling granulosa cell apoptosis, insulin signalling, and OS responses [121].

In PCOS, epigenetic dysregulation is a well-documented contributor to disease pathogenesis [122]. Altered methylation of CYP17, INS, and AMH genes, among others, underpins hyperandrogenism, IR, and follicular dysfunction [123]. If UV filters further perturb these epigenetic networks, they could not only aggravate disease in affected women but also program susceptibility in offspring. This aligns with the ‘developmental origins of health and disease’ paradigm, suggesting that prenatal or periconceptional exposure may predispose daughters to PCOS-like phenotypes [124]. Animal models provide supporting evidence: maternal oxybenzone exposure has been associated with reproductive abnormalities and metabolic dysfunction in progeny [58], echoing features of PCOS. While direct human data remain limited, these findings highlight the need for longitudinal birth cohort studies assessing UV filter exposure and epigenetic biomarkers. Therefore, epigenetic effects represent a critical mechanistic layer linking UV filters to PCOS risk, extending beyond individual women to future generations. This underscores the importance of precautionary exposure management during reproductive years.

## 6. Preclinical and Clinical Evidence

### 6.1. Animal Models: Reproductive Toxicity of Oxybenzone/Octinoxate

Animal models offer pivotal insights into the reproductive toxicity of sunscreen UV filters, as they enable a systematic evaluation of ovarian structure, hormone regulation, and fertility outcomes under controlled exposure conditions [52,114]. Studies in rodents consistently demonstrate that oxybenzone alters ovarian morphology, with findings of follicular atresia, reduced corpus luteum formation, and disrupted estrous cyclicity [75]. These histological changes correlate with decreased estradiol levels, increased testosterone concentrations, and irregular reproductive hormone profiles, which are consistent with the endocrine disturbances observed in PCOS [81]. Octinoxate has similarly been shown to impair ovarian physiology in experimental models. Female rats exposed to octinoxate exhibited increased ovarian weight, elevated androgen levels, and decreased aromatase activity, culminating in disrupted folliculogenesis. Histopathological examinations reveal degeneration of granulosa cells, abnormal follicle development, and impaired luteinization [125]. Importantly, both compounds induce reproductive outcomes consistent with subfertility, including reduced mating success, smaller litter sizes, and delayed puberty onset in offspring [126].

Mechanistic investigations indicate that these toxic effects are mediated through receptor-level perturbations, steroidogenic enzyme disruption, and oxidative stress [52,78]. Mitochondrial dysfunction in granulosa cells and alterations in apoptotic markers have also been reported, providing biological plausibility for UV filter-induced ovarian impairment [78]. Given that PCOS is characterized by similar abnormalities, hyperandrogenism, follicular arrest, and oxidative stress, the convergence of findings in animal models reinforces the hypothesis that oxybenzone and octinoxate exacerbate PCOS-like pathophysiology. While animal studies cannot fully replicate the multifactorial etiology of human PCOS, they highlight dose-dependent and mechanistically coherent reproductive effects. These findings underscore the necessity of incorporating animal data into risk assessments and call for translational studies to evaluate relevance in human populations, particularly among women with PCOS.

### 6.2. In Vitro Studies: Granulosa/Theca Cell Culture, Steroidogenic Disruption

In vitro studies have played a crucial role in elucidating the cellular and molecular mechanisms by which oxybenzone and octinoxate disrupt ovarian function. Cultured granulosa and theca cells provide controlled systems for dissecting the direct effects on steroidogenesis, apoptosis, and receptor signalling [85,94]. Evidence indicates that oxybenzone suppresses aromatase expression and activity in granulosa cells, thereby reducing estradiol synthesis from androgen precursors. This interference disrupts the estrogen-androgen balance necessary for follicular development [48], echoing the intrinsic dysfunction observed in PCOS ovaries. Additionally, oxybenzone exposure promotes OS and mitochondrial dysfunction within granulosa cells, leading to apoptosis and impaired proliferation [56]. These effects compromise the follicular microenvironment, hindering maturation and ovulatory competence. Octinoxate exhibits comparable disruptions: studies reveal downregulation of FSH receptor signalling in granulosa cells and reduced cAMP-mediated transcriptional activity, impairing estradiol production and follicle viability [13]. In theca cells, octinoxate enhances androgen biosynthesis through dysregulated CYP17 activity, further aggravating androgen excess [47].

Both UV filters also exert cross-talk with nuclear receptors beyond ER and AR, including peroxisome proliferator-activated receptors (PPARs), which regulate lipid metabolism and ovarian function [127]. Their interference with PPAR signalling links environmental exposure to broader metabolic dysfunctions relevant to PCOS. Furthermore, evidence of altered expression of apoptotic genes and microRNAs implicates epigenetic mechanisms in cell culture models [59]. Collectively, in vitro data provide robust mechanistic evidence that UV filters directly impair ovarian cell function, disrupt steroidogenesis, and promote PCOS-like endocrine patterns. These findings, aligned with animal studies, strengthen the translational case for considering oxybenzone and octinoxate as contributors to reproductive dysfunction in susceptible women.

### 6.3. Epidemiological Evidence

Epidemiological studies examining human exposure to sunscreen UV filters have largely focused on urinary oxybenzone concentrations, with emerging links to reproductive health outcomes. Large-scale biomonitoring surveys such as NHANES confirm widespread systemic exposure, with women of reproductive age showing the highest levels [128]. Cross-sectional analyses have reported associations between higher urinary oxybenzone and menstrual irregularities, including longer cycle length and increased incidence of oligomenorrhea [129]. These findings parallel PCOS-associated menstrual dysfunction, raising concern about potential interactions. Other studies suggest relationships between oxybenzone exposure and altered reproductive hormone profiles [31,130]. Elevated urinary concentrations have been linked to reduced FSH levels, increased LH/FSH ratios, and elevated testosterone [8]—all endocrine signatures characteristic of PCOS. Associations with reduced antral follicle counts and impaired ovarian reserve have also been described, although findings remain inconsistent across cohorts.

Evidence linking UV filter exposure to clinical fertility outcomes is limited but suggestive. Some studies report reduced fecundability and increased time-to-pregnancy among women with higher oxybenzone levels, while others indicate potential associations with adverse pregnancy outcomes [131]. Data specifically addressing PCOS prevalence or severity remain scarce, representing a significant research gap. Nevertheless, the overlap between observed hormonal alterations and PCOS pathophysiology lends biological plausibility to the concept. Importantly, existing studies are limited by their cross-sectional designs, reliance on self-reported menstrual outcomes, and lack of stratification by PCOS-specific criteria. Despite these limitations, epidemiological evidence aligns with preclinical findings, supporting the hypothesis that oxybenzone and octinoxate may influence ovarian endocrinology and reproductive outcomes in women. Longitudinal, PCOS-focused cohort studies are urgently needed to clarify causal relationships.

### 6.4. Data Gaps: Lack of PCOS-Specific Biomonitoring Cohorts

Significant gaps hinder the evaluation of oxybenzone and octinoxate in PCOS contexts despite robust mechanistic evidence. Most biomonitoring studies address the general population, rarely stratifying by PCOS, despite altered xenobiotic metabolism, adiposity, and oxidative stress, which may modify susceptibility. Evidence remains largely cross-sectional, limiting causal inference. Prospective cohorts specific to PCOS, integrating biomonitoring with reproductive and metabolic phenotyping, are urgently needed. Co-exposure to phthalates, parabens, and bisphenols is rarely assessed, and experimental models often fail to reflect chronic low-dose exposure. Regulatory frameworks also overlook PCOS-specific endpoints, underscoring the need for precision toxicology.

## 7. Mixture Exposures and Real-World Relevance

Human exposure to EDCs rarely occurs in isolation. Sunscreen UV filters such as oxybenzone and octinoxate coexist with phthalates, bisphenol A (BPA), and parabens, chemicals widely used in cosmetics, plastics, and personal care products [132]. Biomonitoring studies consistently detect these compounds in urine, serum, and follicular fluid, confirming ubiquitous co-exposure [6]. Mechanistically, these chemicals converge on overlapping endocrine pathways: phthalates act as anti-androgens, BPA disrupts estrogenic and thyroid signalling, and parabens weakly mimic estrogens. Combined with the estrogenic and anti-androgenic actions of UV filters, these exposures exert additive or supra-additive effects on ovarian function [132,133]. For PCOS women, who already exhibit hyperandrogenism, insulin resistance, and oxidative stress, such chemical mixtures may intensify reproductive and metabolic abnormalities, although PCOS-specific data remain scarce. Importantly, the endocrine-disrupting impact of mixtures is not purely additive but may be synergistic. Low-dose exposures to combinations of oxybenzone, BPA, and phthalates have produced stronger endocrine disruption [134] than that predicted by single-compound studies. Synergistic effects arise through receptor cross-talk, enzyme interference, and overlapping induction of oxidative stress and inflammation, further impairing granulosa cell function and folliculogenesis. For PCOS patients, this cumulative burden may worsen ovulatory dysfunction and metabolic complications, highlighting the need for mixture-based risk assessment. To address this complexity, quantitative mixture modelling offers a valuable approach. Hazard indices, concentration addition, and independent-action frameworks facilitate the estimation of cumulative risk, while machine learning and exposome-wide association studies can identify high-impact chemical combinations associated with PCOS severity. Despite the promise, mixture modelling is underutilized in reproductive toxicology, and PCOS remains underrepresented in such analyses. Integrating biomonitoring with endocrine-metabolic profiling and systems biology models would refine precision risk estimates. Recognizing and modelling mixture effects is therefore essential to capture real-world exposures and inform regulatory policy for women at heightened risk.

## 8. Translational and Clinical Perspectives

Sunscreen UV filters, such as oxybenzone and octinoxate, present a clinical paradox: they are essential for photoprotection, yet they exhibit systemic absorption and have the potential to disrupt the endocrine system. Their estrogenic, anti-androgenic, and steroidogenic enzyme-modulating actions overlap with key mechanisms of PCOS, including hyperandrogenism, anovulation, IR, and oxidative stress [9,130,132]. Women of reproductive age, particularly those with PCOS, are among the highest users of sunscreen products and therefore constitute a subgroup at heightened risk. This raises important questions about whether the use of chemical sunscreens may inadvertently exacerbate reproductive and metabolic dysfunction in this population. A careful risk-benefit appraisal is needed. Photoprotection remains indispensable, but safer alternatives such as mineral-based sunscreens (zinc oxide, titanium dioxide) or newer organic filters with minimal systemic bioavailability should be prioritized in high-risk women. Clinicians across dermatology, gynecology, and endocrinology should provide integrated counselling that balances cancer prevention with reproductive safety, empowering women to make informed choices without inducing alarm. Future translational priorities include the development of ovarian-specific biomarkers, such as follicular fluid or extracellular vesicle signatures, to capture subtle effects of UV filters, alongside PCOS-focused cohorts and exposome-based studies. Such precision frameworks would guide both clinical care and regulatory policy, ensuring that effective photoprotection is achieved without compromising reproductive health.

## 9. Regulatory and Policy Dimensions

The regulation of sunscreen UV filters such as oxybenzone and octinoxate reflects an evolving recognition of their dual role as protective agents against UV radiation and as potential EDCs (Table 2). Regulatory responses have been fragmented across jurisdictions. In the United States, the FDA continues to classify these compounds as permitted sunscreen ingredients but has called for additional safety data, as maximal-use trials have demonstrated systemic absorption exceeding toxicological thresholds [41]. In contrast, the European Union has imposed stricter concentration limits, with the SCCS restricting oxybenzone to 6% and octinoxate to 10% in cosmetic formulations, citing emerging evidence of hormonal interference [43]. Several countries and regions, including Hawaii and Palau, have gone further by banning these filters altogether, primarily on ecological grounds, given their contribution to coral reef bleaching and aquatic toxicity [47]. While these bans were not initiated with reproductive health in mind, they reflect increasing public and policy awareness of the broader environmental and human safety profile of these chemicals. Despite these advances, regulatory frameworks remain insufficiently tailored to reproductive disorders such as PCOS. Current toxicity testing emphasizes fertility and developmental endpoints but neglects disease-specific vulnerabilities such as hyperandrogenism, disrupted folliculogenesis, and insulin resistance. Incorporating PCOS-focused endpoints into regulatory evaluation, alongside encouraging the development and adoption of green chemistry alternatives, would better align public health protection with clinical realities. Such policy innovation is crucial to reconcile photoprotection needs with reproductive safety in vulnerable populations.

## 10. Future Research Priorities and Conclusions

Although mechanistic and toxicological evidence strongly implicates oxybenzone and octinoxate in ovarian disruption, PCOS-specific investigations remain limited. Future research should prioritize large-scale biomonitoring stratified by PCOS status, assessing urinary, serum, and follicular fluid levels to clarify exposure-effect relationships and potential metabolic alterations in this subgroup. Mechanistic studies must extend beyond receptor-level interactions to encompass oxidative stress, adipokine dysregulation, and insulin resistance while incorporating chronic low-dose and mixture exposure paradigms. Exposome-wide approaches using advanced analytics could further delineate co-exposures with phthalates, parabens, and bisphenols. Translational studies, including randomized trials comparing mineral-based sunscreens with conventional formulations in PCOS women, are also needed to provide actionable clinical guidance. Epigenetic investigations exploring DNA methylation, histone modifications, and extracellular vesicle cargo may reveal transgenerational risks.

Taken together, the current evidence indicates that UV filters such as oxybenzone and octinoxate are not biologically inert. Their estrogenic, anti-androgenic, thyroid-disrupting, oxidative, and epigenetic actions intersect with the core mechanisms of PCOS. These interactions may intensify hallmark features of the disorder, including hyperandrogenism, disrupted follicle maturation, and metabolic imbalance. Despite these concerns, most regulatory assessments still overlook PCOS-specific endpoints, treating reproductive risk in broad and generalized terms. This omission leaves a crucial gap in evaluating how such compounds affect women already vulnerable to endocrine disturbances. Bridging that gap will require coordinated efforts, integrating mechanistic research, precision toxicology, and evidence-based policy reform. Only through such integration can the proven dermatological value of photoprotection be balanced with reproductive safety, ensuring that women with PCOS are protected in both health and environment.

## Figures and Tables

**Table 1:** Mechanistic disruption by sunscreen ultraviolet (UV) filters and clinical correlates in polycystic ovary syndrome (PCOS).

Mechanistic Pathway	Oxybenzone/Octinoxate Action	PCOS-Relevant Clinical Correlates	References
Estrogenic and anti-androgenic activity	ER agonism, AR antagonism, and altered sex hormone ratios	Hyperandrogenism, menstrual irregularities, hirsutism	[47,51]
Steroidogenic enzyme interference	Aromatase inhibition, CYP17/CYP19 dysregulation	Elevated androgens, impaired estradiol synthesis, and follicular arrest	[52,53]
Thyroid disruption	Reduced T4, increased TSH, and TPO inhibition	Higher prevalence of subclinical hypothyroidism in PCOS	[26,54]
Oxidative stress and mitochondrial dysfunction	ROS generation, mitochondrial depolarization, apoptosis	Worsened insulin resistance, poor oocyte quality	[55]
Epigenetic reprogramming	DNA methylation, histone modifications, miRNA dysregulation	Transgenerational inheritance of PCOS-like phenotypes	[56,57]

**Table 2:** Clinical and regulatory perspectives on sunscreen ultraviolet (UV) filters in relation to polycystic ovary syndrome (PCOS).

Domain	Current Evidence/Status	Clinical Relevance for PCOS	References
Human biomonitoring	>95% detection of oxybenzone in NHANES; higher in reproductive-age women	Women with PCOS are high consumers of cosmetics. Thus, potentially higher body burden	[32,124]
Epidemiological associations	Linked to altered menstrual cycles, hormone ratios, and diminished ovarian reserve	Overlap with PCOS features (oligomenorrhea, hyperandrogenism)	[66,124]
Preclinical/clinical evidence	Animal/in vitro studies show ovarian toxicity, follicular arrest, and subfertility	Mechanistic convergence with PCOS pathophysiology	[49,52]
Mixture exposure risk	Co-exposure with BPA, phthalates, and parabens results in additive/synergistic disruption	Real-world endocrine burden may exacerbate PCOS severity	[127,129]
Regulatory landscape	FDA: further safety data required; EU: stricter thresholds; bans in Hawaii, Palau	PCOS-specific endpoints absent in toxicological assessments	[40,42]

## Data Availability

No new data were created or analyzed in this study.
